# Bilateral Elevated Macular Lesions

**Published:** 2011-01

**Authors:** Nasi Samiy, Zahra Alami Harandi, Masoud Naseripour, Timothy Y Y Lai

**Affiliations:** Retina Institute of the Carolinas, USA; Associate Professor, Tehran University of Medical Sciences, Tehran, Iran; Professor, Tehran University of Medical Sciences, Tehran, Iran; Associate Professor, Department of Ophthalmology and Visual Sciences, The Chinese University of Hong Kong, China

## CASE PRESENTATION

A 65-year-old woman presented with decreased vision in both eyes of 2 months’ duration. She reported no history of systemic disorders. Visual acuity (VA) was 20/200 in her right and 20/400 in her left eye. Slit lamp examination was unremarkable and intraocular pressure was normal. On fundus examination, an elevated lesion was noted in the macula in both eyes.

**Figure 1:**
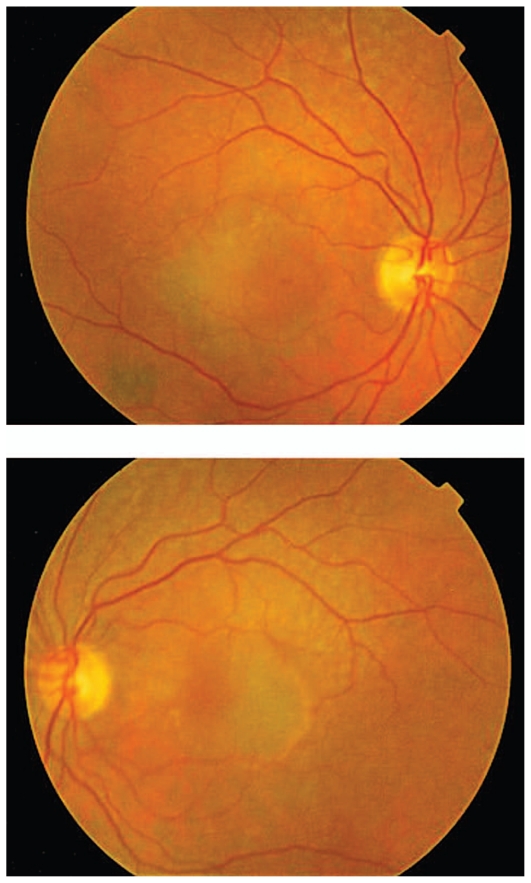
Color fundus photographs of the right and left eyes.

**Figure 2:**
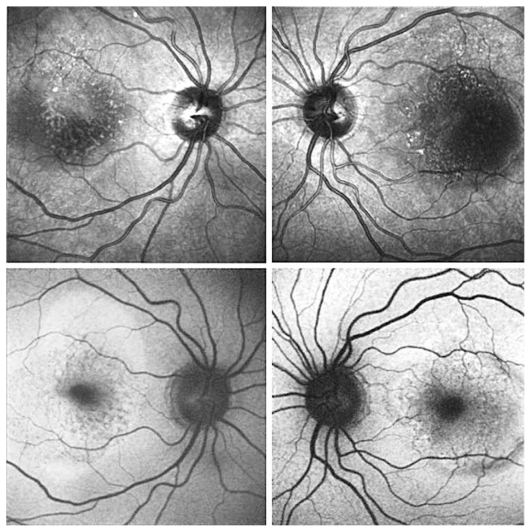
Infrared (upper) and autofluorescence (lower) images of both eyes.

**Figure 3:**
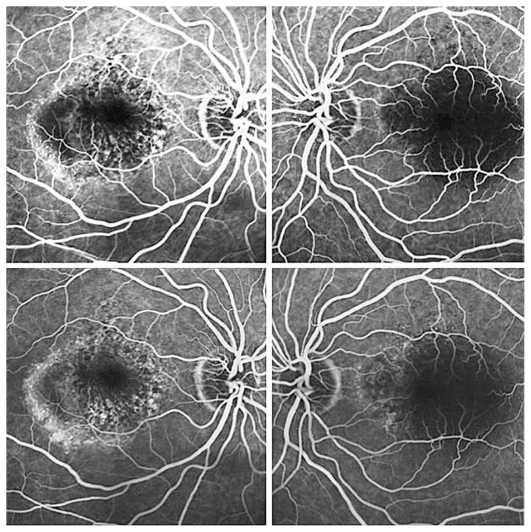
Fluorescein angiograms of both eyes at 0:30 (upper images) and 1:20 (lower images) time frames.

**Figure 4:**
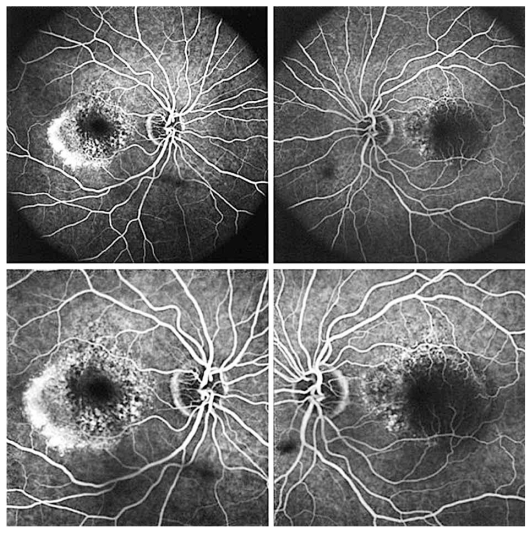
Fluorescein angiograms of both eyes at 2:10 (upper images) and 2:30 (lower images) time frames.

**Figure 5:**
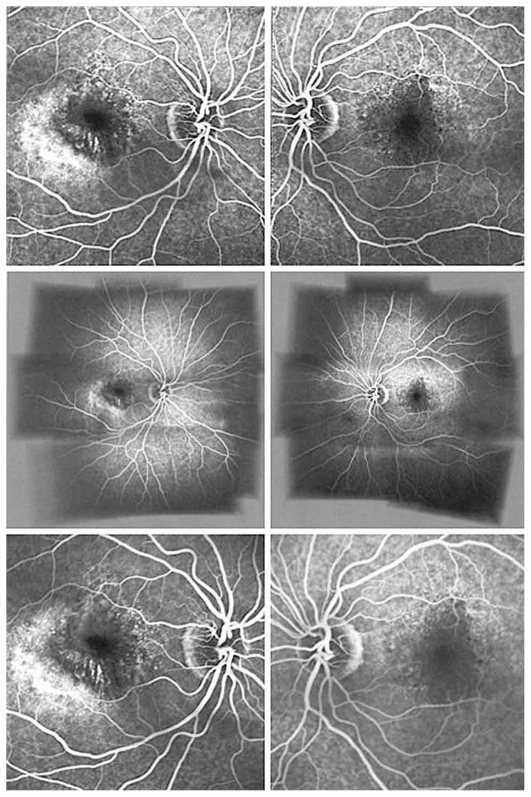
Fluorescein angiograms of both eyes at 3:10 (upper images), 5:30 (middle images), and 7:10 (lower images) time frames.

**Figure 6:**
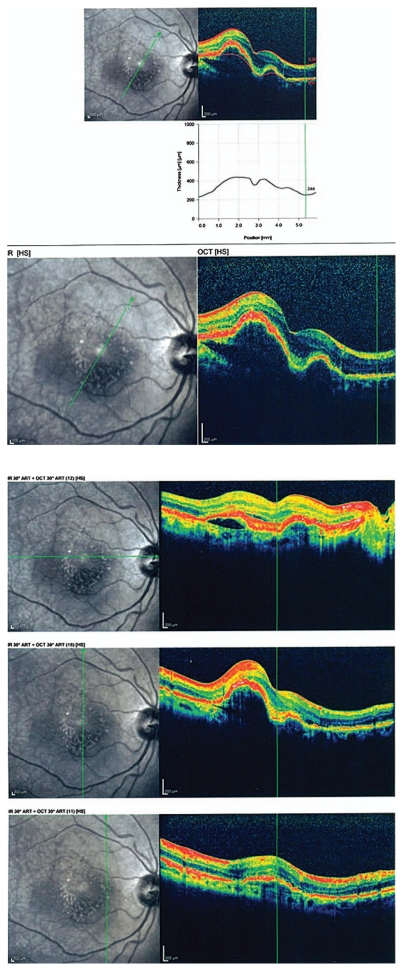
Optical coherence tomography scans of the right eye with different radial scan orientations.

**Figure 7:**
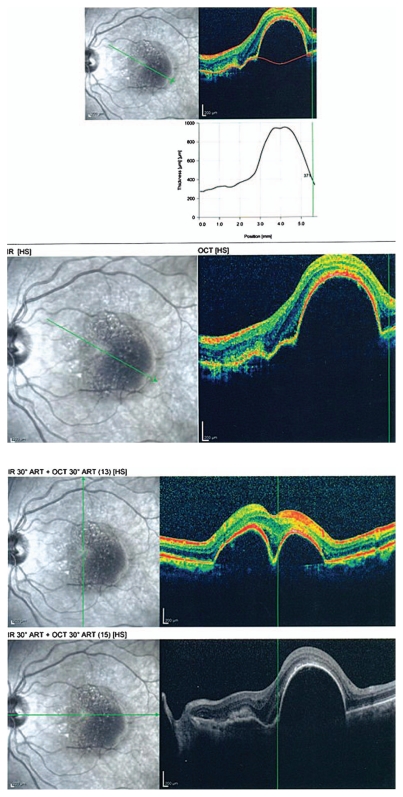
Optical coherence tomography scans of the left eye with different radial scan orientations.

Figures show color fundus (Fig. 1), infrared, and autofluorescence (AF) images (Fig. 2), as well as fluorescein angiography (FA) (Fig. 3 to 5) and optical coherence tomography (OCT) images (Fig. 6 and 7) at presentation. Herein we present the views of four vitreoretinal specialists on the diagnosis and treatment of this patient.

### Nasi Samiy, MD

This patient is a 65-year-old woman with reduced vision of 2 months’ duration, more severe in her left eye. Imaging studies are noteworthy for the presence of retinal pigment epithelium (RPE) detachments and subretinal fluid in both eyes (OU) on OCT (Figures 6 and 7) and occult leakage on FA, OU (Figures 3 to 5). The differential diagnosis would include wet age-related macular degeneration (AMD), central serous retinopathy (CSR), polypoidal choroidal vasculopathy (PCV), and posterior scleritis. Given the patient’s age and gender, I would approach this patient as a case of bilateral wet AMD until proven otherwise.

My first step would be to obtain an indocyanine green angiography (ICGA) which would help with the diagnosis of CSR and PCV. If ICGA is not available, I would start empiric treatment with an intravitreal anti-VEGF (vascular endothelial growth factor) agent.

One issue is whether to treat both eyes in the same session or on separate days. Concerns for endophthalmitis deter me from treating both eyes on the same day. The other potential complication of such management is RPE tear because of the height of the retinal pigment epithelial detachment (RPED). I would certainly discuss this possibility with the patient prior to initiating therapy.

CSR tends to occur in a younger age group but may also present in older subjects. Based on OCT and FA findings, it may be difficult to distinguish CSR from an occult choroidal neovascular membrane (CNVM). Furthermore, the classic “smokestack” finding on FA is seen in only 15% of patients with CSR. One helpful finding is relatively good vision despite the presence of significant subretinal fluid and RPED. However, poor vision, as in this patient, does not preclude a diagnosis of CSR. ICGA can show a more diffuse involvement of the choriocapillaris, with multiple distinct areas of RPED. In this particular patient, it would be helpful to ascertain recent steroid use. The presence of blood would have been helpful in that it rules out CSR and makes a diagnosis of wet AMD or PCV more likely.

ICGA could also be helpful for determining PCV as the etiology. The most helpful finding would be a lattice-like network of fine vessels at the choriocapillaris with bulging ends. These areas would correspond to areas of serosanguineous RPEDs. My treatment of choice for PCV is photodynamic therapy (PDT).

A retinal angiomatous proliferation (RAP) lesion is part of the spectrum of wet type AMD. RAP begins as an intraretinal neovascularization that evolves into a retinochoroidal anastomosis. One can often find intraretinal blood as well as RPED once an anastomotic connection between the RAP and the choriocapillaris has been made. Video ICGA can be particularly useful for delineating a fine choroidal capillary network with a feeder retinal arteriole and a draining retinal venule. My treatment of choice for RAP is the combination of an anti-VEGF agent and PDT.

One should also consider posterior scleritis as a possible diagnosis, however I doubt this to be the case for this patient because of the lack of pain and the absence of vitreous cells on examination.

### Zahra Alami Harandi, MD

This patient is a 65-year-old woman with decreased VA. In the fundus photograph of both eyes there is a cream–green colored lesion in the fovea, 4 to 5 disc diameters in size, with no hemorrhage or hard exudates but with some flecks (Fig. 1). Fundus AF displays faint hyper-autofluorescence of the lesion with areas of hypoautofluorescence (Fig. 2) within it. FA, in the early phase, shows central hypofluorescence with surrounding hyperfluorescence (Fig. 3). In the late phase, the hyperfluorescence becomes more intense and larger (Figures 4 and 5). There is vascularized RPED in both eyes on OCT images (Figures 6 and 7). According to these findings, the most probable diagnosis is occult choroidal CNVM type I, a form of wet AMD.

Patients with neovascular AMD usually present with distortion or loss of central vision. On biomicroscopic examination there is usually serous or hemorrhagic detachment of the neurosensory retina and/or RPE with or without hard exudation. Drusen and RPE changes are often present in the affected or fellow eye. Occult CNVM is divided into two types: (1) fibrovascular RPED (type I), and (2) late leakage of undetermined source (type II). The combination of CNVM and serous RPED has been termed vascularized RPED (V-RPED) and consists of sub-RPE neovascularization associated with a serous RPED.

FA remains, to date, the gold standard for establishing the diagnosis of exudative AMD, and is also required to determine the location, type, size, and degree of activity of the exudative lesion. In fibrovascular RPED, FA shows hyperfluorescence especially 1 to 2 minutes after dye injection, beneath an irregular RPE with either well- or poorly-defined boundaries, and persistent staining or late leakage.

In wet AMD, ICGA is often needed to confirm occult CNVM and the diagnosis of RAP and idiopathic PCV. ICGA is also superior for identifying the vascularized and serous component of V-RPED, as the serous component of an RPED is hypofluorescent while the vascular component is hyperfluorescent.

AF imaging provides information on RPE condition and indirectly, the photoreceptors. In cases of occult CNVM, multiple foci of low AF signals are commonly seen at the site of the lesions which represent small areas of RPE loss. Alternatively more irregular patterns of AF may be present which are followed by growth of a CNVM. AF imaging is useful for following the behaviour of the CNVM and the status of the RPE following treatment. It may help to predict visual outcomes after treatment. It was recently demonstrated that intact foveal AF is associated with better visual outcomes after anti-VEGF therapy. OCT can be used to determine the presence of sub- or intraretinal fluid, and may be useful in the follow up of patients after treatment.

The differential diagnoses of occult CNVM include vitelliform foveomacular dystrophy, chronic CSR, and choroidal tumors. Foveomacular dystrophy can mimic occult CNVM, however, the high intensity AF signal corresponding to the yellow deposits seen in this condition is not observed in AMD.

In the management of vascularized RPED, the efficacy of current treatment options is limited. Studies have shown that PDT and intravitreal triamcinolone acetonide (IVTA) administration do not effectively treat fibrovascular RPED. In a retrospective analysis, Chen et al reported on the use of intravitreal bevacizumab (IVB) in eyes with refractory fibrovascular RPED without any classical components which had previously received intravitreal pegaptanib sodium injections, thermal laser, PDT, or IVTA. This study suggested that bevacizumab may be a good first-line treatment for fibrovascular RPEDs. Interestingly, OCT images showed near-complete resolution of intraretinal and subretinal fluid, without a significant change in the height or area of the RPED itself. Furthermore, patients with these positive OCT changes had an improvement in vision, even without resolution of the RPED. It seems that improvement in vision is attributable to reduction in retinal edema, despite the lack of change in RPED size.

RPE tears are known to develop spontaneously, as well as after conventional laser therapy, PDT, and anti-VEGF therapy. The most likely mechanism of RPE tear is mechanical contraction of the CNVM underneath the RPED. In RPE tear, foveal sparing and continued suppression of neovascular activity appear to contribute to visual preservation. Some patients experience improved or preserved vision with additional anti-VEGF therapy despite the RPE tear.

In conclusion I would recommend IVB administration for this particular patient despite the risk of inducing RPE tears.

Suggested Readings1ChenEKaiserRSVanderJFIntravitreal bevacizumab for refractory pigment epithelial detachment with occult choroidal neovascularization in age-related macular degenerationRetina2007274454501742069610.1097/01.iae.0000249574.89437.402LadasIDKotsolisAIPapakostasTDRouvasAAKaragiannisDAVergadosIIntravitreal bevacizumab combined with photodynamic therapy for the treatment of occult choroidal neovascularization associated with serous pigment epithelium detachment in age-related macular degenerationRetina2007278918961789101310.1097/IAE.0b013e3180ca9ad93ChanCKLinSGRetinal pigment epithelial tear after ranibizumab therapy for subfoveal fibrovascular pigment epithelial detachmentEur J Ophthalmol2007176746761767195010.1177/1120672107017004324Axer-SiegelREhrlichRRosenblattIKramerMPrielEYassurYPhotodynamic therapy for occult choroidal neovascularization with pigment epithelium detachment in age-related macular degenerationArch Ophthalmol20041224534591507866110.1001/archopht.122.4.453

## Masoud Naseripour, MD

Based on the provided information, this 65-year-old woman demonstrates bilateral RPED associated with occult CNVM in the context of exudative AMD.

Although RPED may be associated with different chorioretinal diseases, including CSR, malignant hypertension, and choroidal inflammatory diseases such as the Vogt-Koyanagi-Harada syndrome, the most common condition is AMD. The literature shows that up to 10% of all patients with AMD present with RPED. The pathogenesis of RPED and its clinical subtypes are beyond the scope of this case presentation, however causes of this entity include: stage II and III RAP, PCV, and a marginally situated occult CNVM.

Clinical signs suggesting the presence of a CNVM underlying an RPED include: subretinal fluid, lipid exudates, presence of hemorrhage, and chorioretinal folds. Fundus photography of both eyes in this patient shows bilateral serous RPED surrounded by an orange pigment ring, a possible indicator of chronicity, in the left fundus (Fig. 1).

AF imaging would be of great value for evaluating the integrity of the outer retina and assessing therapeutic interventions for exudative AMD in the era of anti-VEGF treatment. Small foci of reduced AF signals observed in both AF frames could be compatible with an occult CNVM (Fig. 2). The different quality of AF images between the eyes may be due to several factors including focusing, intensity of flashes, etc.

Regarding infrared (IR) frames, although occult CNVM shows poorly demarcated areas of scattered IR increase, the presence of associated lesions such as fibrin coagulates, drusen and RPE alterations may change the appearance of IR frames. The appearance of RPED on IR is determined by its shape, melanin content, and composition of sub-RPE fluid. Furthermore, the focus plane of scanning laser ophthalmoscopy (SLO) can affect the appearance of RPED. A dark core on the IR frame of the left eye shows that SLO has been focused deep to Bruch’s membrane, in contrast to the right eye IR frame, in which the presence of a bright spot indicates that the focal plane is near the apex of the serous detachment and that light has been scattered by the RPE (Fig. 2). Nevertheless, higher turbidity of sub-RPE fluid in the left eye cannot be ruled out.

FA shows pinpoint areas of speckled hyperfluorescence (more evident in the right eye) in the early phase (Fig. 3) followed by larger areas of hyperfluorescence at the margin of the lesion in late phase angiograms, particularly in the right eye (Figures 4 and 5). The source of leakage cannot be discerned from earlier phase FA. In such a case, ICGA is particularly useful for delineation of occult CNVM, and sometimes the condition may be reclassified into well-defined or classic CNVM. Several studies have confirmed the superiority of ICGA for detecting CNVM in patients with exudative AMD associated with RPED.

Simultaneous IR (Fig. 2) and OCT images (Figures 6 and 7) of the patient show RPEDs with hyporeflective spaces at the base in both eyes, associated with a small amount of subretinal fluid at the margin of the RPED in some frames. Although the increased noise of OCT images has decreased their resolution, the presence of small hyperreflective dots with thickening and breaks in the RPE represents CNVM, particularly at the margin of the RPEDs.

When interpreting OCT images in patients with RPED and neovascular AMD, one must keep in mind that RPED goes beyond the area of obvious hyperfluorescence on FA and this may cause some discrepancy between FA and OCT imaging.

Because of the limited options for treatment of AMD patients with occult CNVM associated with RPED and severe vision loss, and the possibility of serious postoperative complications such as RPE tear, the management of these patients remains controversial.

The incidence of RPE tear in different studies ranges from 1.8% to 27%, these include natural history studies and reports on eyes undergoing different treatment modalities. As suggested by different studies, the natural course of patients with RPED and neovascular AMD is characterized by progressive visual loss. Over time, more fibrovascular tissue associated with chorioretinal atrophy develops and ultimately results in a fibrotic disciform scar. This process can be accelerated by RPE tears and subretinal hemorrhage. In addition, in such patients, the second eye is frequently involved (such as this patient) and a bilateral disciform macular scar often develops as the disease progresses.

Most authors believe that despite the risk of post-intervention visual loss, a high rate of stabilization in VA and morphological changes can be achieved with current treatment modalities, including PDT and intravitreal injection of anti-VEGF agents and/or steroids. Although all available therapeutic procedures have not yet been compared in a large randomized clinical trial, combination therapies such as intravitreal anti-VEGF with steroids or PDT, as well as PDT with IVTA, may be more effective than monotherapy for eyes with RPED associated with CNVM.

Identifying eyes at risk of RPE tear before initiating any treatment is very important. These include patients with advanced age, a large irregular vascularized RPED on FA (uneven filling) and/or a large and elevated RPED on OCT, presence of small areas of RPE thinning or small holes along the margins of the RPED, and vitreomacular traction on OCT.

In my opinion, in this particular case, a bilateral intravitreal injection of bevacizumab plus dexamethasone or triamcinolone 2 to 3 weeks apart (to see if any possible RPE tear will develop in the first eye) could be a good and cost-effective option. If no post-treatment complications arise, multiple intravitreal injections should be considered.

Suggested Readings1YannuzziLAHope-RossMSlakterJSGuyerDRSorensonJAHoACAnalysis of vascularized pigment epithelial detachments using indocyanine green videoangiographyRetina19941499113751860710.1097/00006982-199414020-000032Zayit-SoudrySMorozILoewensteinARetinal pigment epithelial detachmentSurv Ophthalmol2007522272431747280010.1016/j.survophthal.2007.02.0083ShimaCGomiFSawaMSakaguchiHTsujikawaMTanoYOne-year results of combined photodynamic therapy and intravitreal bevacizumab injection for retinal pigment epithelial detachment secondary to age-related macular degenerationGraefes Arch Clin Exp Ophthalmol20092478999061930844110.1007/s00417-009-1067-94LommatzschAHeimesBGutfleischMSpitalGZeimerMPauleikhoffDSerous pigment epithelial detachment in age-related macular degeneration: comparison of different treatmentsEye (Lond)200923216321681919731810.1038/eye.2008.4255ChiangAChangLKYuFSarrafDPredictors of anti-VEGF-associated retinal pigment epithelial tear using FA and OCT analysisRetina200828126512691862872410.1097/IAE.0b013e31817d5d036ChangBYannuzziLALadasIDGuyerDRSlakterJSSorensonJAChoroidal neovascularization in second eyes of patients with unilateral exudative age-related macular degenerationOphthalmology199510213801386909777710.1016/s0161-6420(95)30860-3

## Timothy Y Y Lai, MD, FRCS

The fundus photographs of this 65-year-old woman demonstrate the presence of a well-demarcated RPED involving the macula in both eyes (Fig. 1). Early (Fig. 3) and late phase FA images (Figures 4 and 5) show RPE window defects around the RPED with minimal leakage, suggesting the disease has a chronic nature. Fundus AF imaging shows multifocal and localized areas of reduced autofluorescence in the central macula (Fig. 2). These findings are nonspecific and may represent abnormal metabolism of RPE cells. In the paracentral macula, AF shows mildy increased autofluorescence which may be associated with a previous episode of exudative neurosensory retinal detachment. Spectral domain OCT images in both eyes confirm the presence of RPED and also demonstrate mild intraretinal thickening and subretinal fluid accumulation (Figures 6 and 7). OCT images in the left eye, demonstrate a localized hyperreflective area at the level of the RPE nasal to the RPED which may represent a CNVM or deposition of abnormal material such as protein or vitelliform substances (Fig. 7). In view of the absence of hyperfluorescence on FA and the lack of increased autofluorescence at the corresponding location, the material is more likely to be fibrinous protein material.

RPED can be classified into four subtypes including serous RPED, fibrovascular RPED, hemorrhagic RPED, and drusenoid RPED. Common causes of RPED include AMD, PCV, and CSR. In patients with RPED, additional assessment with ICGA would be very useful to detect the presence of CNVM associated with neovascular AMD or polypoidal lesions in patients with PCV. Moreover, ICGA can provide better evaluation of the choroidal circulation in order to detect dilated choroidal vessels and choroidal hyperpermeability which are commonly seen in patients with CSR.

In this patient, since OCT showed the presence of RPED associated with subretinal fluid and macular thickening, intravitreal injections of anti-VEGF agents such as ranibizumab or bevacizumab might be useful to improve vision by reducing RPED and subretinal fluid. My routine practice is to inject three loading doses of an anti-VEGF agent at monthly intervals and perform serial OCT imaging to assess the treatment response. Studies have demonstrated that anti-VEGF therapy is effective in treating serous RPED associated with neovascular AMD. If the treatment response to initial anti-VEGF therapy is poor, ICGA is strongly recommended, as the disease might be caused by PCV, and combination therapy using PDT with verteporfin and intravitreal anti-VEGF agents may be useful for such cases. In cases of serous RPED associated with CSR, PDT using standard or half-dose verteporfin has been demonstrated to be useful; such treatment can reduce RPED size and improve vision.

Suggested readings1CasswellAGKohernDBirdACRetinal pigment epithelial detachment in the elderly: classification and outcomeBr J Ophthalmol198569397403240865910.1136/bjo.69.6.397PMC10406162LommatzschAHeimesBGutfleischMSpitalGZeimerMPauleikhoffDSerous pigment epithelial detachment in age-related macular degeneration: comparison of different treatmentsEye (Lond)200923216321681919731810.1038/eye.2008.4253LaiTYChanWMLiuDTLukFOLamDSIntravitreal bevacizumab (Avastin) with or without photodynamic therapy for the treatment of polypoidal choroidal vasculopathyBr J Ophthalmol2008926616661835626510.1136/bjo.2007.1351034LaiTYChanWMLiHLaiRYLiuDTLamDSSafety enhanced photodynamic therapy with half dose verteporfin for chronic central serous chorioretinopathy: a short term pilot studyBr J Ophthalmol2006908698741659766610.1136/bjo.2006.090282PMC18571715KimKSLeeWKPhotodynamic therapy with verteporfin for avascular serous pigment epithelial detachment in elderly KoreansRetina20103093991969670210.1097/IAE.0b013e3181b094a1
